# High-resolution circa-2020 map of urban lakes in China

**DOI:** 10.1038/s41597-022-01874-6

**Published:** 2022-12-03

**Authors:** Chunqiao Song, Xingan Jiang, Chenyu Fan, Linsen Li

**Affiliations:** 1 Key Laboratory of Watershed Geographic Sciences, Nanjing Institute of Geography and Limnology, Chinese Academy of Sciences, Nanjing, 210008 China; 2 Changwang School of Honors, Nanjing University of Information Science & Technology, Nanjing, 210044 China; 3 University of Chinese Academy of Sciences, Beijing, 100049 China; 4 College of Surveying and Land Information Engineering, Henan Polytechnic University, Jiaozuo, 454000 China

**Keywords:** Hydrology, Limnology, Environmental impact

## Abstract

Urban lakes provide important ecological services to local communities, such as flood mitigation, biodiversity, and recreation. With rapid urbanization, urban lakes are significantly affected by socio-economic development and urgently need attention. Yet there is still a lack of datasets that include tiny urban lakes on a global or national scale. This study aims to produce a high-resolution circa-2020 map of urban lakes (≥0.001 km^2^) in China. The 10-m-resolution Sentinel-2 imagery and a simple but robust water extraction method was used to generate waterbodies. The accuracy of this national-scale dataset was evaluated by comparing it with manually sampled urban units, with the average accuracy of 81.85% in area and 93.35% in count. The database totally inventories 1.11 × 10^6^ urban lakes in China, with a net area of ~2.13 × 10^3^ km^2^. Overall, the spatial distribution of urban lakes in China showed strongly heterogeneous characteristics. This dataset will enhance our understanding of the distribution pattern of China’s urban lakes and contribute to better ecological and environmental management as well as sustainable urban development planning.

## Background & Summary

Lakes comprise a critical component of global hydrological and biogeochemical cycles and provide essential resources for human and ecosystem well-being^1–5^. In recent years, accelerating climate changes and intensive anthropogenic disturbances have exerted pronounced influences on global lakes. In return, they impact the regional climate and available water resources for human society^6–10^. As lakes serve as an integrative indicator of climate and environmental changes as well as human interventions within their drainage basins, knowledge of the abundance and distribution of lakes is thus very crucial for a wide range of socioeconomic, political, and scientific interests.

Urban lakes are widely deemed as artificial ecosystems, usually small in size and shallow in depth^11^. The environmental quality of life in urban areas is directly related to the access to urban lakes for their different functions. Through their ecological and functional characteristics, urban lakes can provide numerous ecosystem services closely related to human well-being, such as fishing, flood mitigation, reeding, phosphorus, and nitrogenous retention, biodiversity, maintaining species population and habitats, regulation of the urban microclimate, as well as educational and recreational services^12–16^. As urban lakes can most effectively emit longwave radiation to cool the surface and efficiently consume shortwave radiation through evaporation^17,18^, the urban design embedded with them can provide cooling benefits and reduce human exposure to heat stress^19,20^. In Europe, the climate change mitigation and adaptation plans adopted by urban administrations are intensively based on green (vegetation cover) and blue (waterbodies) solutions^21,22^. The case study on two of the largest lakes of Bucharest (i.e., Herăstrău and Morii) confirm that the extent of the thermal influence depends both on the characteristics of the water bodies^23^. These small urban lakes also differ from those larger and more intensively investigated lakes in terms of physical and biogeochemical conditions and processes^24^. For example, they tend to be high in dissolved organic matter, dissolved CO_2_, and nitrogen concentration^25–28^, and therefore more significantly impact methane production^29,30^ and CO_2_ efflux^31,32^.

Urban lakes, whether natural or man-made, are very different from other non-urban lakes: they are shallow, relatively small, highly artificial, and often hypertrophic, yet more people come into contact with them^11^. Given the direct impacts of human activities in urban areas, urban lakes are considered as one of the most vulnerable freshwater ecosystems in the world^33^. Globally, 55% of the world’s population lived in urban areas in 2018, and this proportion is projected to be 68% by 2050^34^. Over the past several decades, urbanization, a major way of the Earth’s surface alteration. Rapid urbanization has been posing increasingly significant threats to the urban scenery waterbodies^35^, and has resulted in various environmental and ecological problems^36^. At present, the conservation of urban lakes is facing severe challenges^37–39^, such as the continuous reduction in the lake area, water eutrophication or pollution^40,41^, and the degradation of riparian vegetation and biotopes^42,43^, which hinder the development of the economy and destructed urban landscape. Hence, there is a pressing need to conduct intensive research to grasp the information on the urban lake distribution and characteristics in the context of rapid urbanization to benefit the informed decisions for lake managers and to guide future city development better.

China has experienced an accelerated expansion of cities since the 1978 implementation of economic reforms^44,45^, which accompany ever-greater demands on services that nearby and distant aquatic ecosystems provide^46^. Triggered by rapid urban sprawl and population growth, Chinese urban lakes have been seriously influenced by intensive land-use changes. Consequently, the conflict between rapid urbanization and the maintenance of urban lakes in China urgently needs to be addressed^47^. There has been a long-standing concentration of research efforts on areal variations of the national-scale lakes (>1 km^2^) in China^4,9,48–53^, but with incomplete coverage of the urban lakes. The pioneering studies on urban lakes in China involve a wide range of topics, for example, the distribution and variability of urban lakes and the associations with land-use changes^33,47,54^, water quality deterioration due to anthropogenic disturbances^55–57^, and the effects of water bodies on urban microclimates and matter^58–60^. Yet these studies mostly focus on the individual or a few lakes in a local area or at a single-city level (e.g., Wuhan City in Central China)^61,62^. For the national-scale study, Xie *et al*.^63^ first performed a remote sensing investigation of the spatiotemporal variations of urban lakes in China’s 32 major cities between 1990 and 2015 and revealed that the urban lakes experienced drastic shrinkage and landscape fragmentation in the past 25 years. Overall, the spatial distribution, abundance, and landscape characteristics of the lakes in all the urban areas of China yet remained largely unknown.

The objective of this study is to provide a high-resolution map of urban lakes (surface water area ≥0.001 km^2^) in China. First, we used high-resolution Sentinel-2 imagery acquired in circa-2020 (2019–2021) and a waterbody index method that was designed for urban areas to generate preliminary waterbody extraction results in China at a 10-m spatial resolution on Google Earth Engine. After converting to vectors, the waterbody extraction results were filtered based on the spatial relationships with urban area boundaries to remove non-urban waterbodies. Then, combined with sub-meter resolution historical satellite imagery, we manually removed non-lake water bodies, such as rivers, paddy fields, fish ponds, etc., and edited the missing urban lakes. Using high-resolution remote sensing images and a waterbody index method that was designed for urban areas, the dataset mapped the waterbodies of urban lakes in China, and defined three kinds of waterbodies as urban lakes according to the spatial relationships between lakes and urban boundaries with the GUB data for reference. This dataset is mainly designed to fill the gaps in a complete inventory of urban lakes in China as well as to better understand the distribution and characteristics of urban lakes in the context of rapid urbanization.

## Methods

### Study data

#### Sentinel-2 data

Sentinel-2 includes two polar-orbiting satellites (Sentinel-2A and -2B, respectively launched in June 2015 and March 2017, https://sentinel.esa.int/web/sentinel/missions/sentinel-2). The revisit period for a single satellite is ten days, and the combined use of double satellites can reach 3–5 days. The multispectral imager (MSI) carried by the Sentinel-2 satellite includes 13 spectral bands from visible light, near-infrared, short wave infrared, of which the spatial resolutions ranging from 10 m to 60 m^64^. In the Google Earth Engine (GEE) platform, Sentinel-2 Level-1C data is the atmospheric surface reflectance data produced after orthophoto correction and fine geometric correction, and Sentinel-2 Level-2A data has been processed with atmospheric correction based on L1C data. In this study, we selected Sentinel-2 Level-2A data to extract the waterbody. The Red-Green-Blue bands and the NIR band with high resolutions of 10 m were used to produce a 10 m resolution dataset of urban lakes in China.

#### Global urban boundaries dataset

The Global Urban Boundaries (GUB) dataset provides a physical boundary of urban areas^65^. Li, *et al*.^65^) combined the kernel density estimation method, the urban growth model, and the morphological method to generate the urban boundary from 1 km impervious surface area (ISA) data aggregated from the global artificial impervious area (GAIA). Finally, the small holes which may be caused by green space or water were filled to retrieve the final boundaries. The high spatial resolution of the dataset and its global coverage makes it popular and widely used^66,67^. At present, the GUB data is available in seven typical years (1990, 1995, 2000, 2005, 2010, 2015, and 2018). We chose the dataset of 2018 as the reference for urban boundaries to define urban lakes. The dataset can be accessed from http://data.ess.tsinghua.edu.cn.

#### World imagery wayback

The World Imagery Wayback is a project conducted by ESRI which is aimed to provide high-resolution satellite and aerial imagery for most parts of the world. It provides users with access to the different versions of World Imagery created from 2014 to now, and every item can be accessed directly in ArcGIS. Although it has a high update frequency, it does not update images of the entire global regions or different zoom levels every time, which means that the latest products may also include historically acquired imagery. So, in most cases, the newest imagery is more desirable and typically preferred. In this study, we used this high-resolution imagery for manual visual interpretation and accuracy evaluation. We chose the latest version (2021-12-21) for reference when eliminating those which do not belong to urban lakes from the initial water extraction results by manual visual interpretation. The high-resolution data can be accessed from https://www.arcgis.com/home/item.html?id=534b917ee7c7472aa416f4b2f8b24f80.

### Production of urban lakes

#### Definition and conceptual diagram of urban lakes

**Fig. 1:**
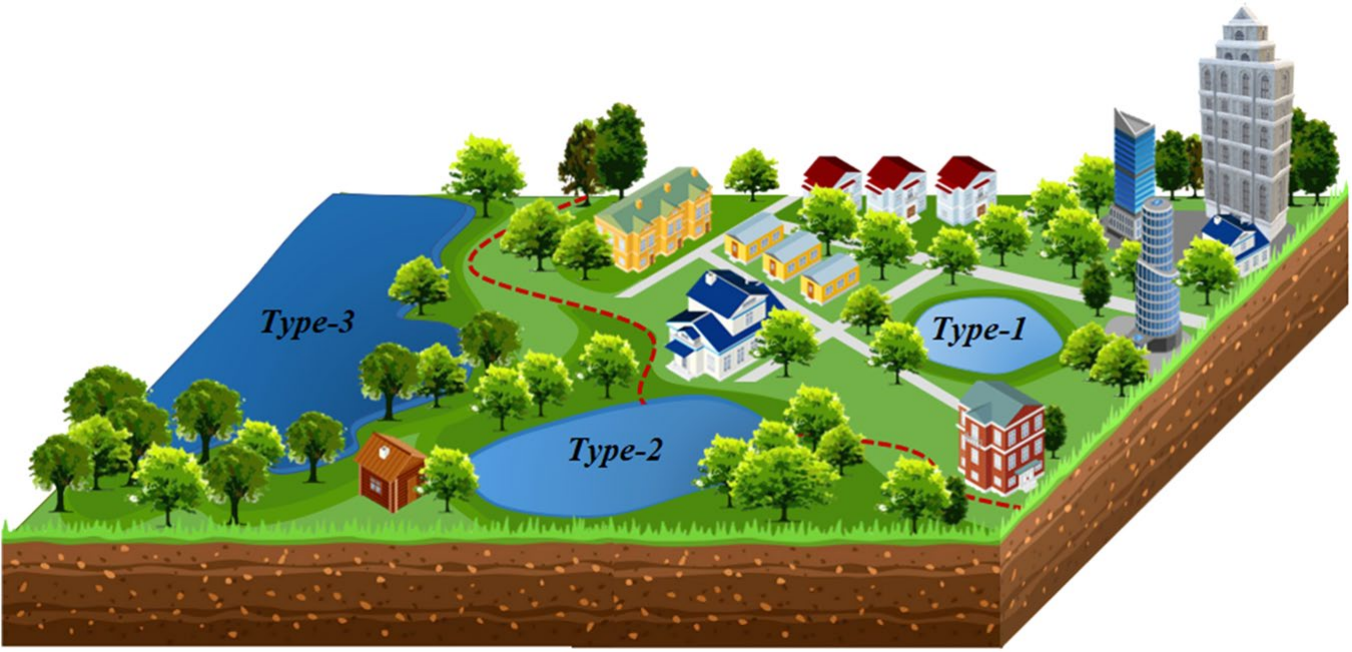
The conceptual diagram of three kinds of urban lakes where the brown dashed line represents Global Urban Boundaries.

In this study, we defined three kinds of water bodies as urban lakes according to the spatial relationships between lakes and urban boundaries with the GUB data for reference (see Fig. 1). First, the lakes (Type-1) which completely fall in the extent of GUB were the most common and typical (e.g., Kunming Lake in Beijing City, Xuanwu Lake in Nanjing City, etc.). Second, the lakes are not completely contained in the scope of GUB but intersect with the boundary (Type-2). This kind of lake is located on the periphery of the city or town. The third kind of lakes, which are especially large and adjacent to principal cities, do not intersect with GUB but are well known to the public as urban lakes (Type 3). In this study, there are a total of 14 lakes of this type inventoried in the dataset. Considering its particularity, we separate them in a single data layer from the first two kinds of urban lakes. Thus, these 14 urban lakes are not included in the statistical analysis of the result part of this article and are only described in the part of “Usage Notes”.

#### Production process of the circa-2020 map of urban lakes

In this study, we used a Two-Step Urban Water Index (TSUWI) method^68^ to detect urban waterbody, which was designed for water extraction in urban areas based on high-resolution imagery. TSUWI is composed of two remote sensing indices, Urban Water Index (UWI) and Urban Shadow Index (USI). UWI is designed to effectively distinguish water and urban shadows from other non-aqueous types, while USI is designed to remove the urban shadows contained in the UWI extraction results. TSUWI refers to the combination of UWI and USI. If both indices are greater than the threshold, it can be considered as waterbody, and the recommended threshold of these two indexes is both 0. So, in this study, we considered that pixels with UWI and USI greater than 0 are water bodies. The two indices are calculated as follows.

1 $$UWI=\frac{G-1.1\times R-5.2\times NIR+0.4}{| G-1.1\times R-5.2\times NIR| }$$

2 $$USI=0.25\times \frac{G}{R}-0.57\times \frac{NIR}{G}-0.83\times \frac{B}{G}+1.0$$

where B, G, and R represent the blue, green, and red bands of Sentinel-2, respectively. NIR is the Sentinel-2 near-infrared band. These bands are all of the 10-m spatial resolution.

To produce the circa-2020 map of urban lakes in China, we selected Sentinel-2 images with a cloud coverage assessment of less than 20% in 2019 – 2021 and used their ‘QA60’ bands to remove the cloud. These images were placed into collections and then spatially overlapped into a composite image, and each pixel represented the median value of this pixel in all images. After calculating UWI and USI from the composite imagery, pixels with both UWI and USI greater than 0 were considered as the preliminarily extracted water bodies. All these steps were completed on GEE (see the code at 10.6084/m9.figshare.20583558)^70^.

**Fig. 2:**
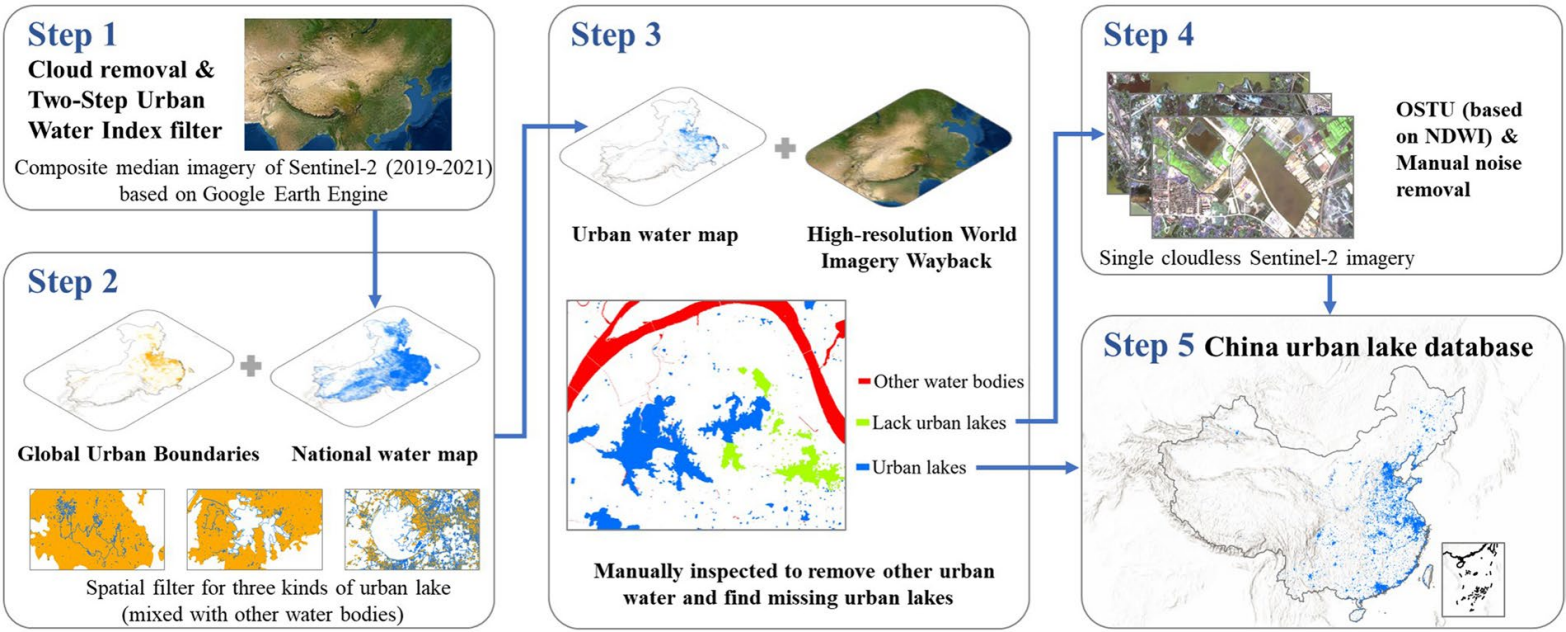
Working flow procedures of the circa-2020 map of urban lakes.

Based on the preliminary waterbody extraction data, we screened the results according to the definition of urban lakes. By overlapping with the World Image Wayback imagery, we manually removed non-lakewater bodies (e.g., rivers, fish farms, etc.) within or near the urban areas. Despite the strict quality control, a small amount of noise may still exist due to the complex environment of urban elements and uncertainties in the GUB data. We eventually keep the lakes with an area of more than 10 pixels (0.001 km^2^) as the smaller ones tend to be more neglected in the quality assurance. In the process of manual inspection, there were a few urban lakes that had not been detected in the initial mapping results due to the extremely turbid water conditions or imagery quality. We recorded these locations and screened the local single-scene cloudless imagery. Then we extracted these missing urban lakes by the OSTU method^69^ or manual digitization and added them back to our quality-control results. The working flows as described above are illustrated in Fig. 2.

#### Accuracy evaluation by comparing with the data from manual digitization

In order to better understand and quantitatively evaluate the accuracy of our dataset, we randomly selected 9 square grids with a size of about 5 km in urban areas in different regions of China and manually digitized the water bodies in them (considering the small number of water bodies in some grids, we scaled some of them up in equal proportion). Finally, urban lakes with an area over 0.001 km^2^ were selected from the manual digitization results and then compared with our urban lake dataset on high-resolution imagery. We summed the area and quantity of urban lakes in each grid, respectively, and cross-evaluated our dataset from these two aspects.

## Data Records

Based on the methods introduced above, we produced the national inventory of Chinese urban lakes by synthesizing all available Sentinel-2 images acquired in 2019–2021. In this dataset, there are 1.11 × 10^6^ urban lakes (over 0.001 km^2^) in China, and the net surface water area exceeds 2.13 × 10^3^ km^2^. The China urban lake dataset (CULD) is available at *figshare* repository (10.6084/m9.figshare.20583558) in the ESRI shapefile format and a description document^70^, which contains three vector data layers: “*China_Urban_Lake_Type1_&_Type2.shp*”, “*China_Urban_Lake_Type3.shp*”, and “*China_Urban_Boundary.shp*”. These data layers are all referenced in the Geographic Coordinate System of “GCS_WGS_1984” with the datum of “D_WGS_1984”. The “*China_Urban_Lake_Type1_&_Type2.shp*” data layer includes 110,698 urban lakes belonging to Type 1 or Type 2 (as illustrated in Fig. 1). In the “*China_Urban_Lake_Type3.shp*” layer, 14 Type-3 large urban lakes are organized separately. The “*China_Urban_Boundary.shp*” data layer provides the polygons of urban areas that are used to judge the spatial relationships with urban lakes, as introduced in the section of “Methods”.

## Technical Validation

### Qualitative evaluation by comparing with historical high-resolution image

**Fig. 3:**
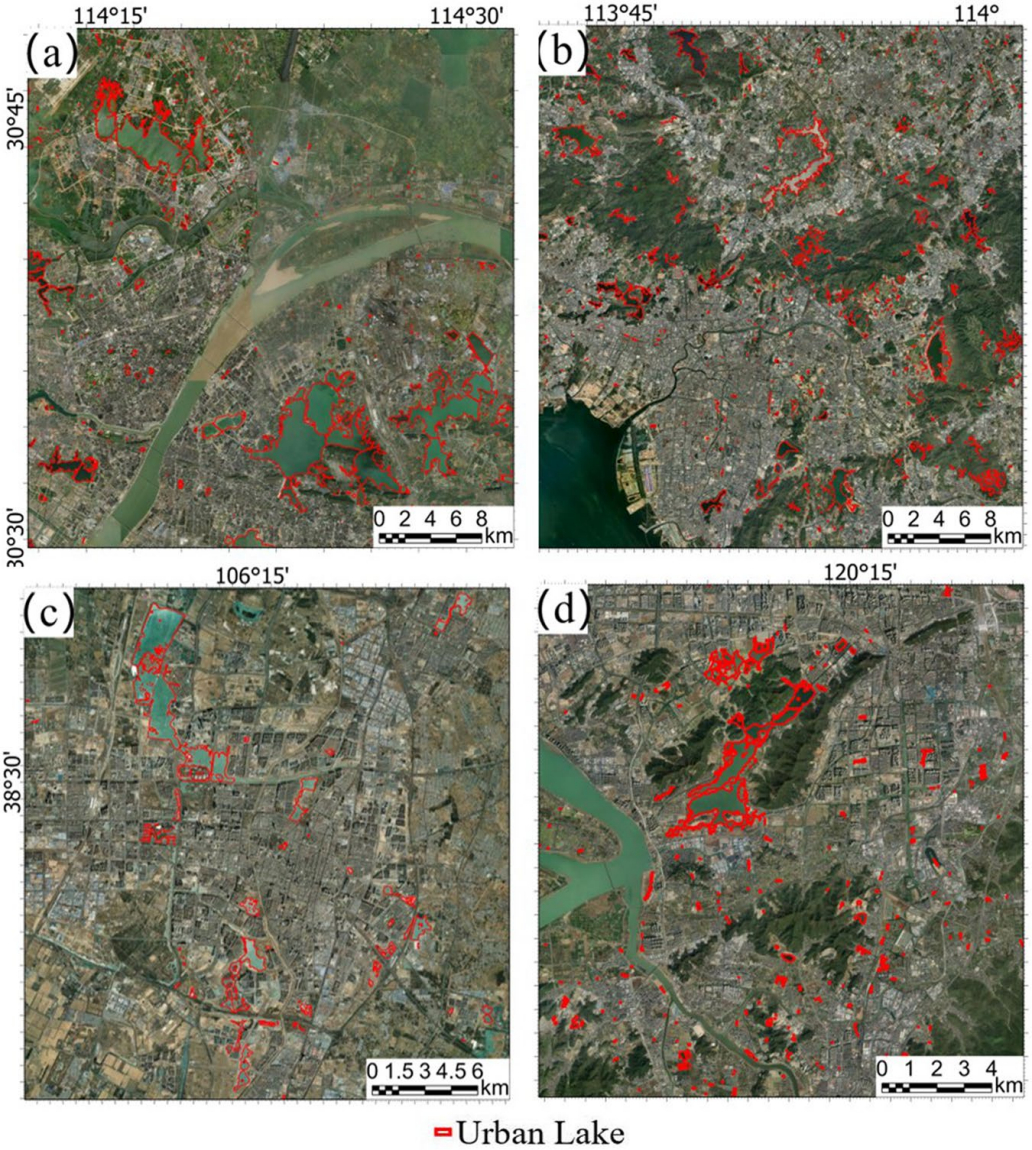
The comparison of the China urban lake database and high-resolution imagery. (**a**) Part of Wuhan; (**b**) Part of Shenzhen and Dongguan; (**c**) Part of Yinchuan; (**d**) Part of Hangzhou.

We selected four typical urban areas in different locations in China and superimposed our dataset with high-resolution historical imagery (Fig. 3). For large lakes, the lake shoreline depicted by our dataset matches the actual lake boundary very well. Those lakes with relatively small areas, for example, the magnitude of 0.001 km² that cannot be found at a low zoom level of high-resolution historical imagery, are also paid attention to without obvious omission errors and they account for a large proportion in the count. The possible mapping errors of these small lakes may mainly lie in the vague division boundary of urban and suburbs, for example, in the northeast of Fig. 3a and centers of Fig. 3b,d.

### Cross-evaluation of the urban lake database with manually digitized data

**Fig. 4:**
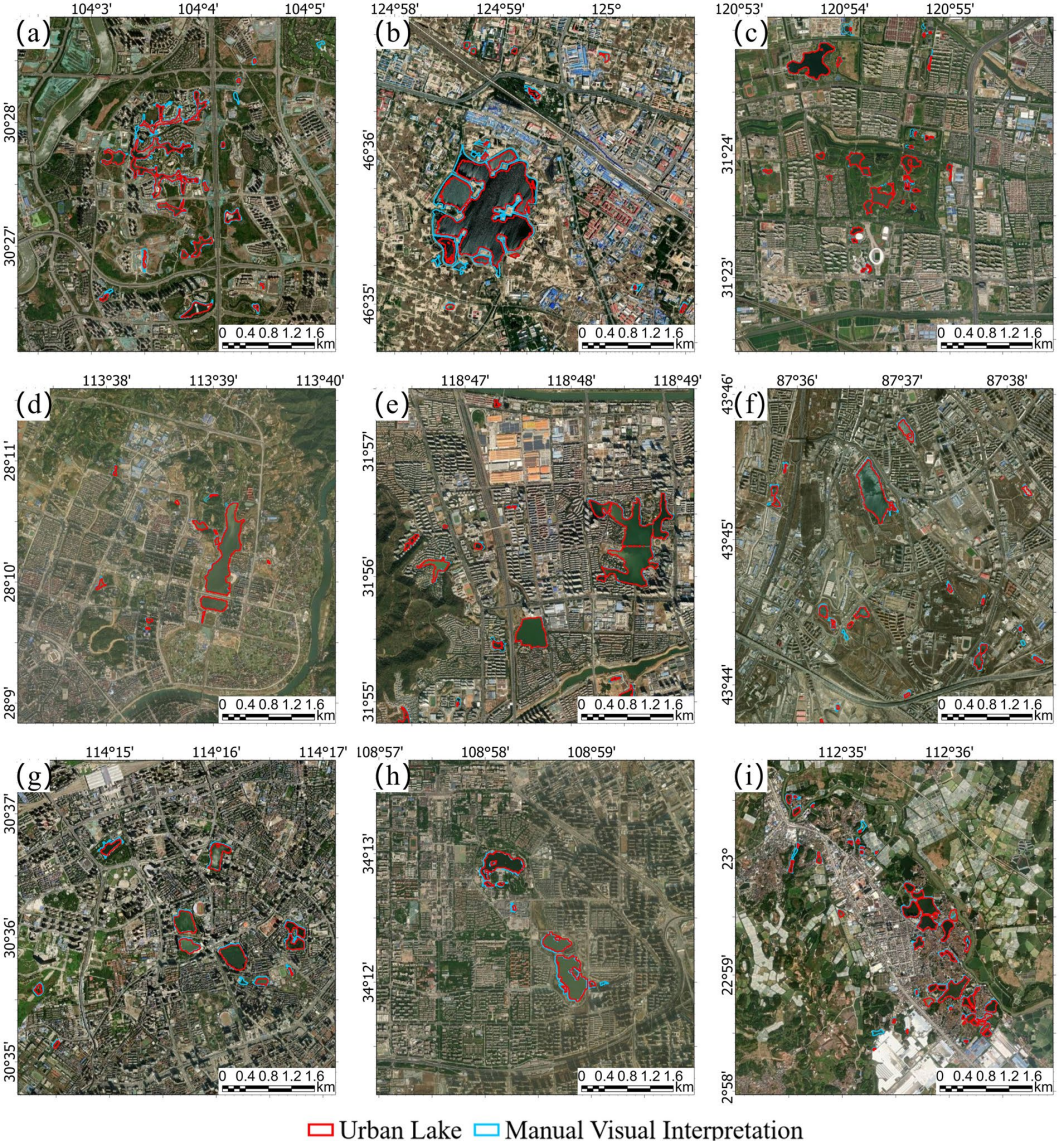
The comparison of China’s urban lake database and manual digitization results on high-resolution imagery in nine sampling areas. (**a**) Part of Chengdu; (**b**) Part of Daqing; (**c**) Part of Kunshan; (**d**) Part of Liuyang; (**e**) Part of Nanjing; (**f**) Part of Wulumuqi; (**g**) Part of Wuhan; (**h**) Part of Xian; (**i**) Part of Zhaoqing.

Figure 4 shows the comparison of China urban lake database and manual digitization results on high-resolution imagery. The two lake layers are in good agreement for all the nine sampling areas of China in both the lake presence and extent. Only for a few cases, the long and narrow waterbodies embedded in the building complex may have some deviations in the lake boundary match (Fig. 4a,b). Considering that the manual digitization results were produced based on the high-resolution imagery at the sub-meter level while our lake dataset was produced based on the 10-m resolution Sentinel-2 imagery, the error from the small waterbody mapping due to the mixing pixel influences is expected and acceptable.

**Fig. 5:**
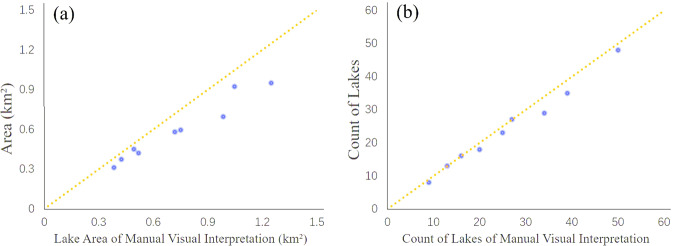
Accuracy evaluation of Chinese urban lake database and manually digitized results in area and count. (**a**) scatter plot for lake area; (**b**) scatter plot for lake count.

Figure 5 illustrates the comparison scatter plot of the areas and numbers of the total urban lakes for each sampling areas derived from the national urban lake dataset and the validation data manually digitized from high-resolution imagery. It is clear to see a good match of our mapping results with the validation data. Overall, the urban lakes mapped from Sentinel-2 images may slightly underestimate the areas than the reference data, as shown in Fig. 5a. The average accuracy of the urban lakes of the area in the nine sampling areas is 81.85%, with the highest accuracy of 91.85% in Liuyang and the lowest accuracy of 70.69% in Chengdu (Figs. 4a,d,5a). When comparing the count of urban lakes, our dataset performs well, and the average accuracy is 93.35% (Fig. 5b). Overall, as a ten m-level product, the national dataset has high accuracy and is acceptable.

### Comparison with existing global or national lake data sets

**Fig. 6:**
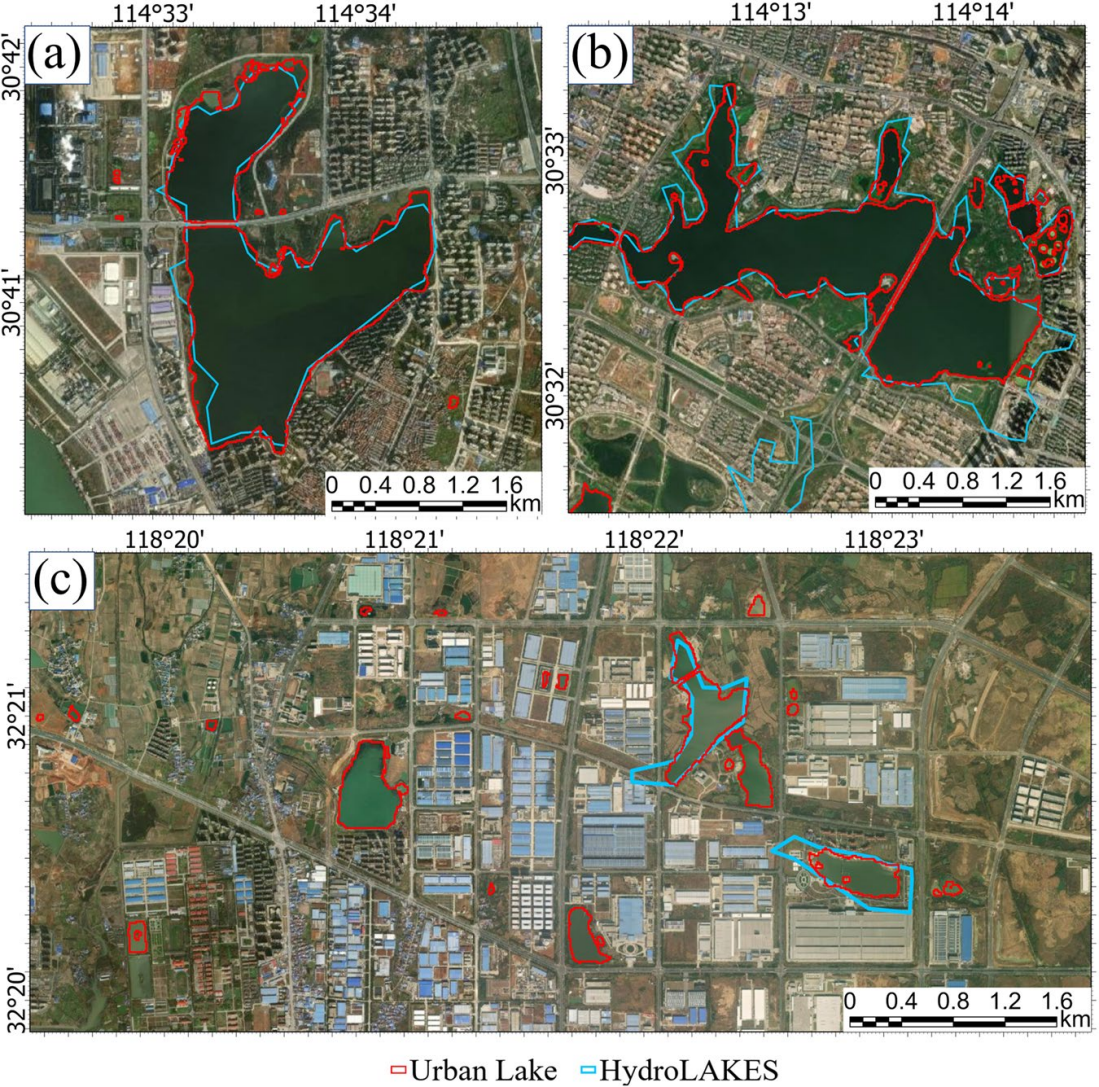
The comparison of China urban lake database and HydroLAKES based on high-resolution imagery in a typical sampling area.

**Fig. 7:**
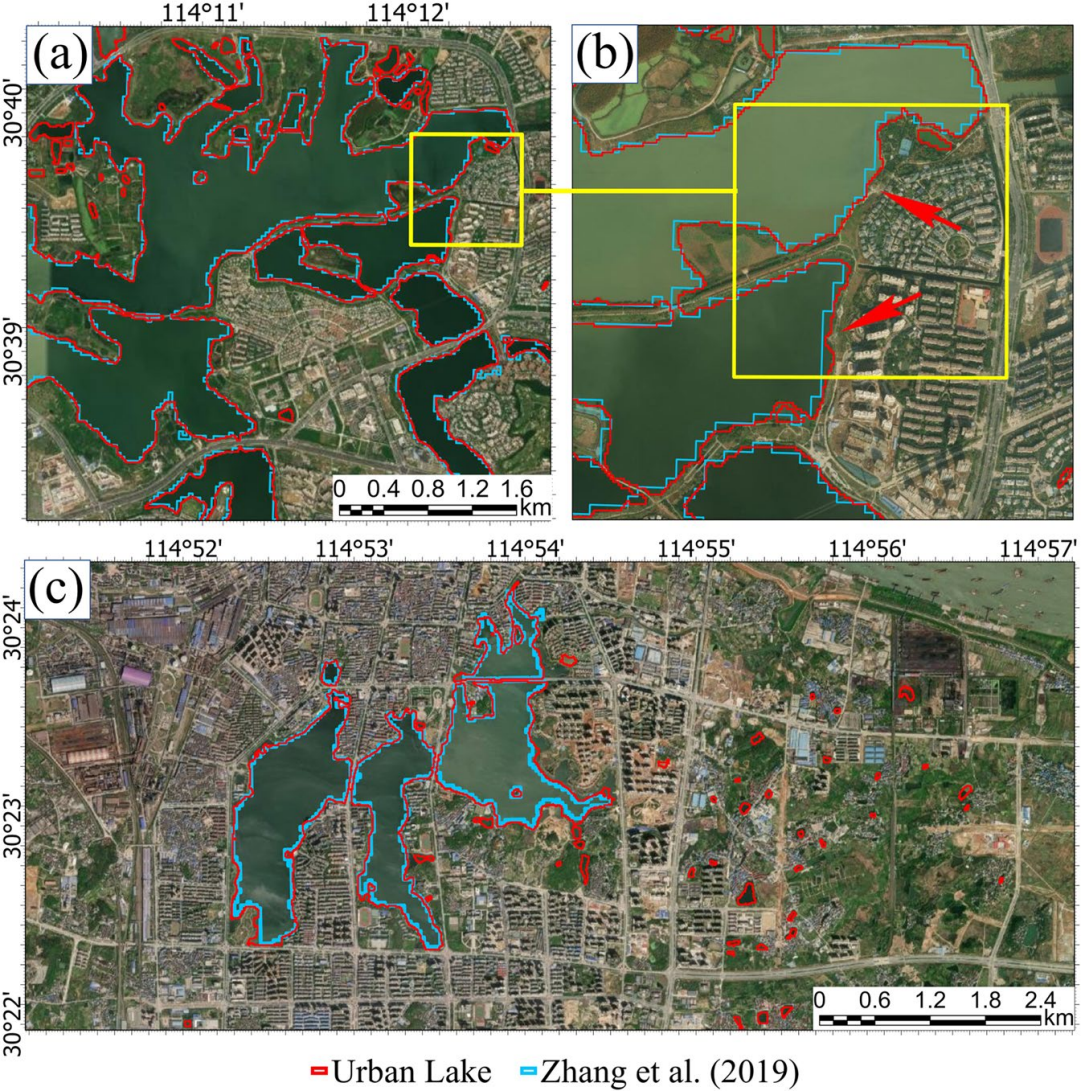
The comparison of China’s urban lake database and Zhang *et al*.^51^ based on high-resolution imagery in a typical sampling area.

The inventory of lake distribution is an interesting topic, which has been addressed by many prior studies on both the global and national scales. HydroLAKES^71^ is one of the most famous global lake data sets. Figure 6 shows its results in Chinese urban areas and compares them with our dataset. Obviously, the data accuracy of HydroLAKES in the urban areas is much lower than the mapping results presented in this study. Although both the two datasets include large urban lakes, the HydroLAKES data has some obvious biases in the outline of lake boundaries (Fig. 6a) and “omission” or “commission” errors (Fig. 6b,c). On one hand, the HydroLAKES only include lakes larger than 10 ha (0.1 km^2^), thus may ignore many small urban lakes are inventoried in our dataset. On the other hand, the HydroLAKES data was produced by compiling multiple sources of historical datasets, such as the SRTM Water Body Data (SWBD) that depict the lake status in February 2000. Therefore, most of the “omission” or “commission” errors may be caused by the rapid land use changes in the urban areas (Fig. 6b,c). To better evaluate the urban lake dataset, we also inspect the uniformity of our mapping results with the national-scale lake (>1 km^2^) dataset of the year 2020 (update after the paper publication) in China^51^. As shown in Fig. 7, both our dataset and Zhang, *et al*. ^51^) show satisfactory accuracy in mapping lakes larger than 1 km^2^. Similar to the comparison illustrated in Fig. 6, there are many small urban lakes missing in the prior dataset (Fig. 7c). Besides, the lake boundaries of this study dataset mapped from 10-m Sentinel-2 imagery can depict the lake shoreline more precisely than that previously derived from Landsat and MODIS data.

## Usage Notes

### Details on Type-1 and Type-2 urban lakes in China

**Fig. 8:**
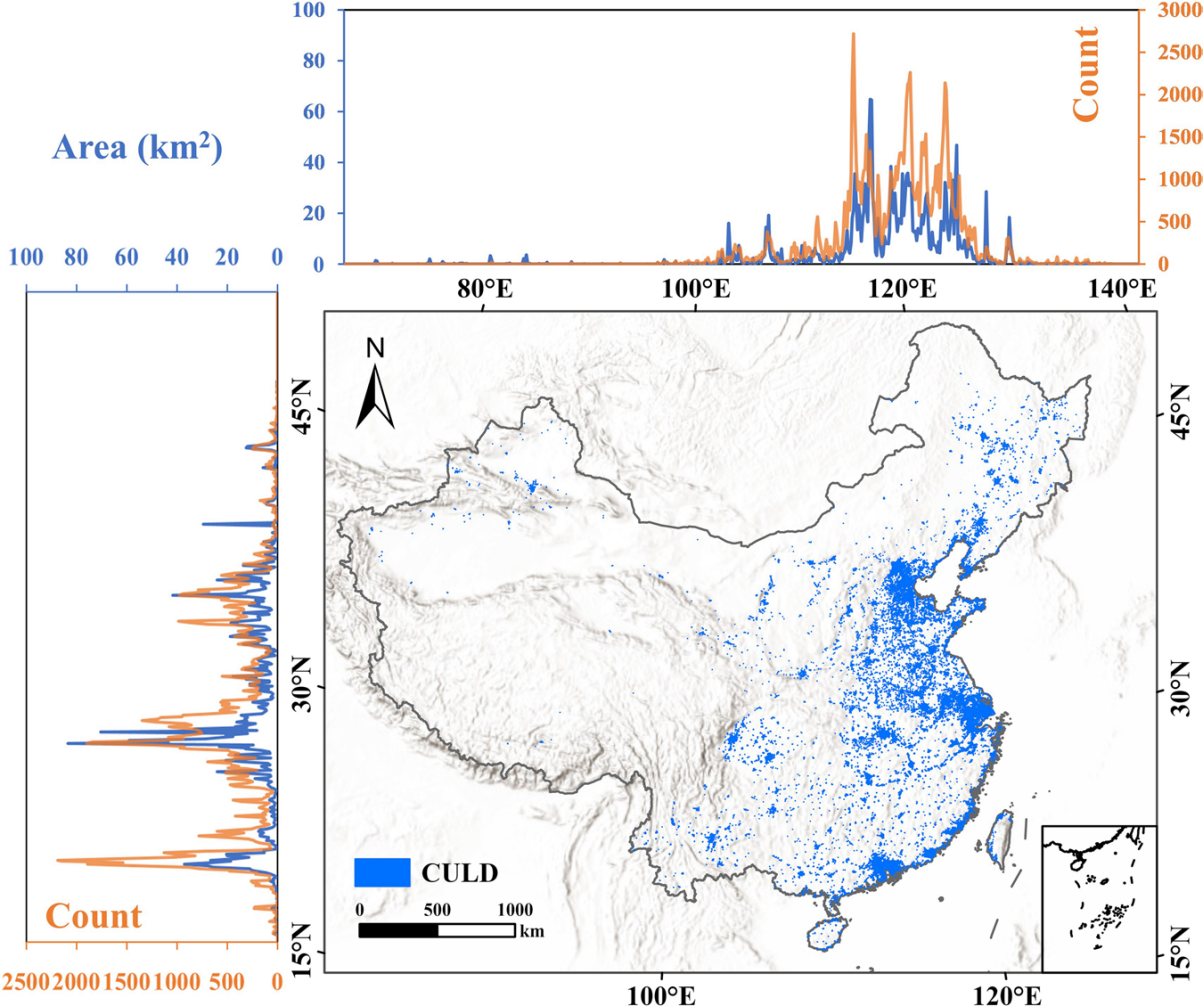
The distribution of urban lakes in China. The area and count of urban lakes by degrees are summed based on 0.1° × 0.1° square grid.

Figure 8 shows the spatial distribution map and latitudinal and longitudinal statistics of urban lakes that belong to Type 1 or Type 2 as illustrated in Fig. 1. Overall, these urban lakes show strong spatial heterogeneous aggregation levels, more in the east and south and less in the west and north of China. The most significant concentrations can be observed in China’s top three urban agglomerations, the Guangdong-Hong Kong-Macao Greater Bay Area, the Yangtze River Delta area, and the Beijing-Tianjin-Hebei area. In the latitudinal and longitudinal patterns, the region where the longitude ranges from 110°E to 125°E and the latitude ranges from 20°N to 35°N accounts for a large proportion of the area and quantity of urban lakes in China. In sharp contrast, from 100°E to the west, many regions have no urban lakes at all, or the number of urban lakes is very small, which is consistent with the arid climate and low economic level.

### Details on Type-3 urban lakes of China

**Fig. 9:**
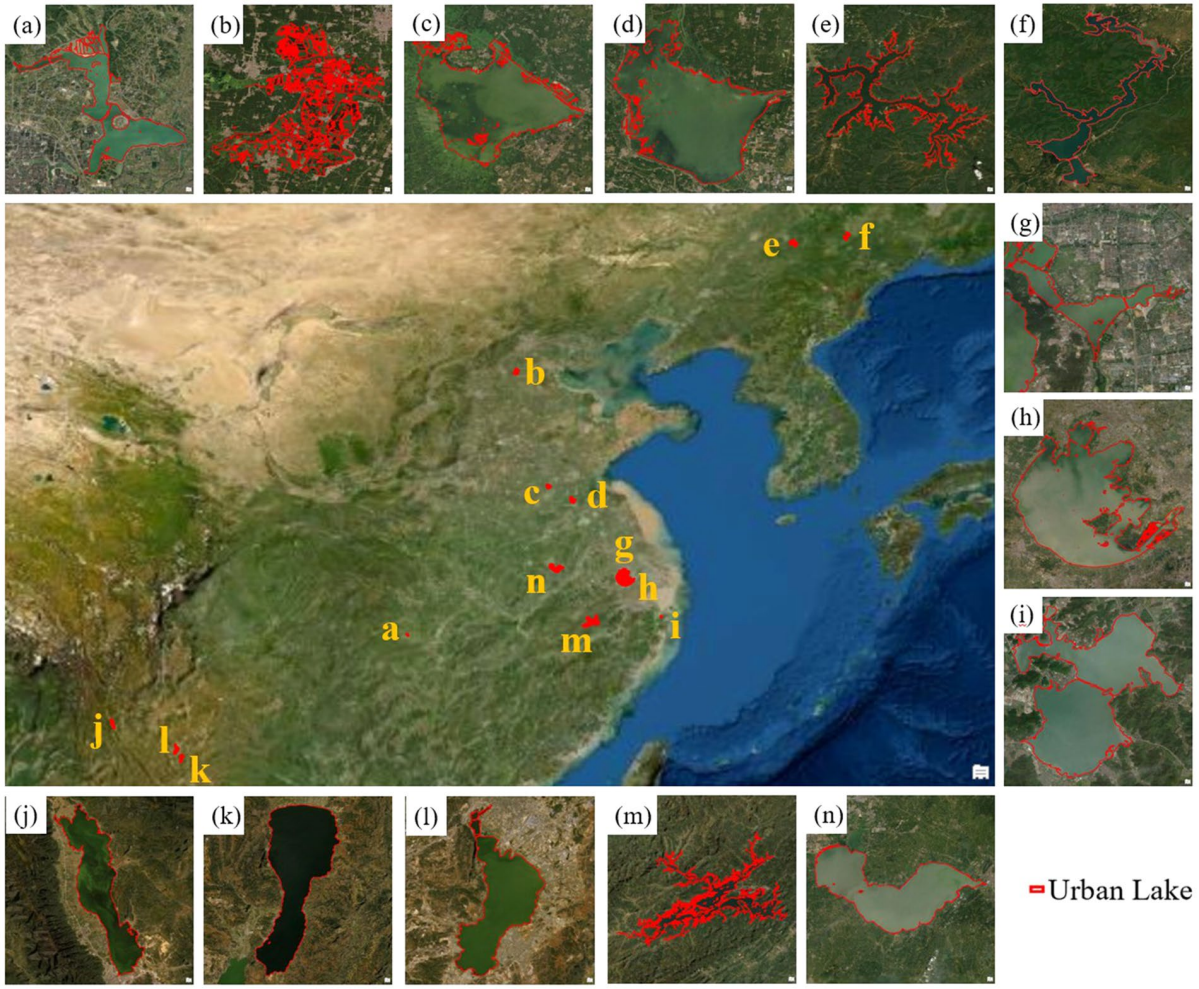
The 14 Type-3 urban lakes that are well known and have a large area around the city. (**a**) Liuye Lake; (**b**) Lake Baiyangdian; (**c**) Lake Weishan; (**d**) Lake Luoma; (**e**) Lake Songhua; (**f**) Lake Jingpo; (**g**) Lake Lihu; (**h**) Lake Taihu; (**i**) Lake Dongqian; (**j**) Lake Erhai; (**k**) Lake Fuxian; (**l**) Lake Dianchi; (**m**) Lake Qiandao; (**n**) Lake Chaohu.

Besides these small urban lakes introduced above, there is another type (Type 3) of urban lakes that are well-known as tourist attractions by the population in the adjacent cities. In this data layer, we total inventory 14 lakes (see Fig. 9). In general, they have large inundation areas and are mostly concentrated in East China. Table 1 shows the basic information of these lakes. The largest lake among them is Lake Taihu with an area of over 2000 km^2^, and the smallest lake among them is Lake Lihu, which has an area around 7 km^2^ and is close to Lake Taihu. Although these lakes may be distant from surrounding cities, they have many ecological or economic functions and play important roles in local communities. For example, in East China, where the terrain is flatter, these lakes, including Lake Taihu, Lake Weishan, and Lake Luoma, provide freshwater resources and convenience for agricultural and recreational activities in the surrounding area. In southwest China where the terrain is undulating, Lake Erhai, Lake Dianchi, and Lake Fuxian are all famous tourist destinations. Of course, all these lakes have the fundamental functions of regulating runoff and water storage and supporting ecosystem wellbeing.

**Table 1:** Basic information of the 14 Type-3 urban lakes.

Name	Latitude (°)	Longitude (°)	Province	City	Area (km^2^)
Lake Taihu	31.20	120.19	Jiangsu	Suzhou/Wuxi	2312.33
Lake Chaohu	31.57	117.53	Anhui	Hefei	779.07
Lake Qiandao	29.56	118.95	Zhejiang	Chunan	461.44
Lake Dianchi	24.82	102.69	Yunnan	Kunming	297.82
Lake Erhai	25.79	100.20	Yunnan	DaLi	243.52
Lake Luoma	34.09	118.19	Jiangsu	Suqian	235.98
Lake Fuxian	24.52	102.89	Yunnan	Yvxi	214.67
Lake Weishan	34.61	117.25	Shandong	Jining	143.33
Lake Songhua	43.65	126.83	Jilin	Jinlin	136.53
Lake Baiyangdian	38.88	116.00	Hebei	Xiongan	82.79
Lake Jingpo	43.90	128.93	Heilongjiang	Mudanjiang	80.48
Lake Liuye	29.09	111.74	Hunan	Changde	19.41
Lake Dongqian	29.77	121.66	Zhejiang	Ningbo	18.62
Lake Lihu	31.52	120.25	Jiangsu	Wuxi	7.04

### Limitations of the China urban lake dataset

**Fig. 10:**
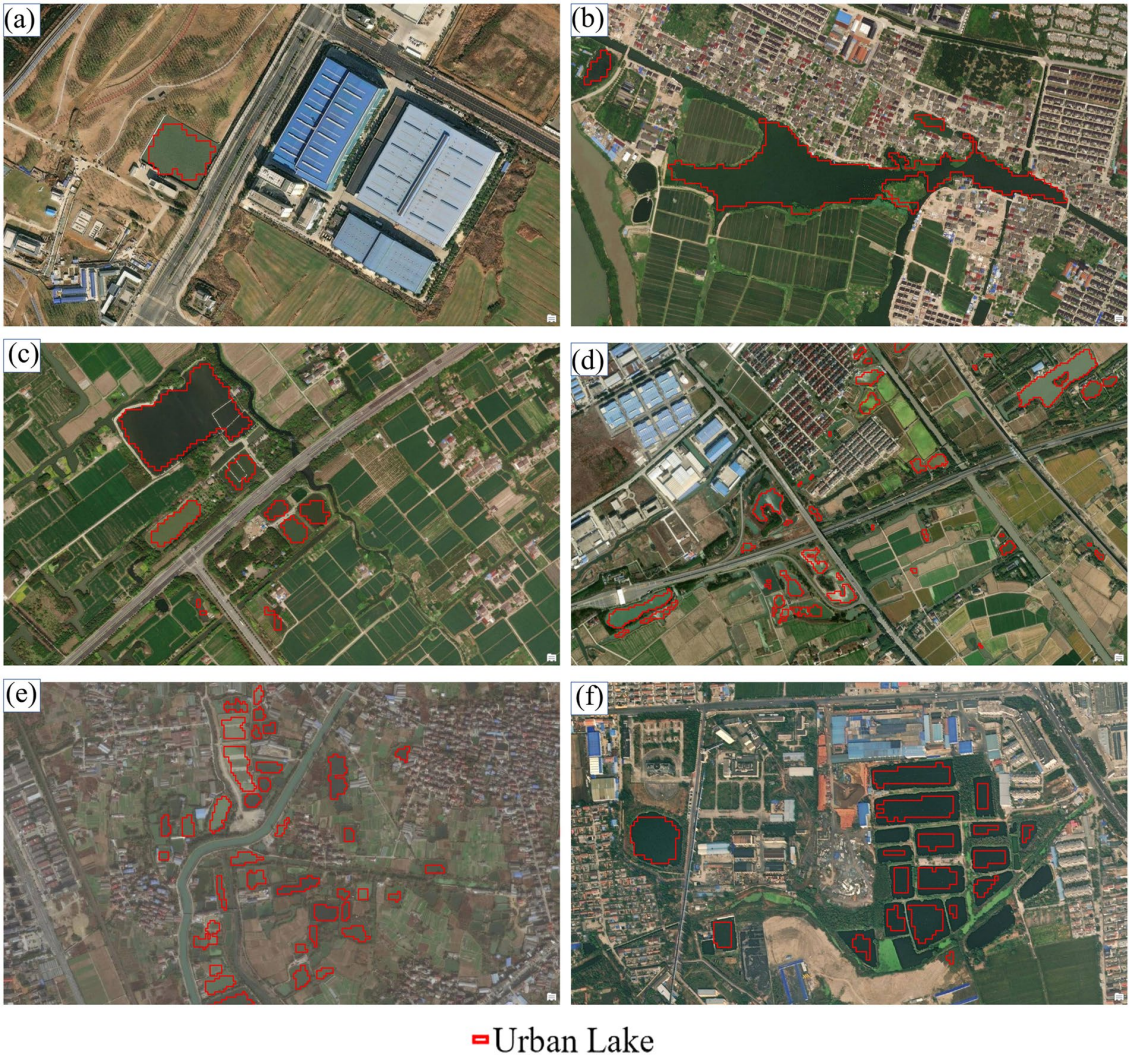
Some confusing water bodies mapped as urban lakes. (**a**) Artificial water bodies located near construction sites, factories, or residential areas; (**b**) The open waters connected to rivers or channels; (**c**–**f**) Regular water bodies possibly used for aquaculture, farming, or similar functions.

In this study, we utilized the GUB urban area data combined with the reference to high-resolution imagery to define and identify the urban lakes. However, it is difficult to guarantee the inerrant distinguishment of lakes from other water bodies as the urban areas actually have no explicit spatial boundary with rural areas or suburbs. Figure 10 shows some confusing water bodies within or intersecting the GUB boundary, which are close to the urban impervious layers and were inventoried in this study. Figure 10a represents some artificial reservoirs that are located near buildings, factories, or residential areas. Figure 10b represents some of the open waters connected to rivers. Figure 10c–f represents those which is regular waterbody possibly used for aquaculture, farming, ponds, or similar functions.

**Fig. 11:**
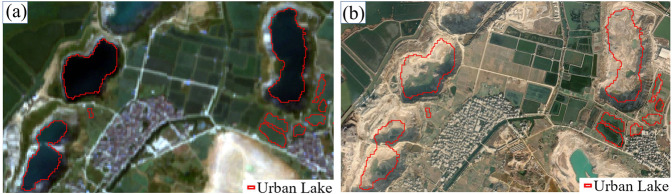
The rapid changes of urban areas which lead to the inconsistence between the Sentinel-2 imagery and high-resolution imagery. (**a**) The median-composite image of Sentinel-2 and the boundaries of urban lakes; (**b**) The high-resolution imagery used for manual visual interpretation and the boundaries of urban lakes from our mapping.

Urban areas are undergoing rapid development in China, with highly frequent land-use changes. Despite that we used the medium composite for each band of all images acquired during 2019–2021, when comparing the mapping result of water extraction with the high-resolution imagery, there still exist a few changes out of our detection and induce uncertainty in the manual visual interpretation. Figure 11 shows the difference between our dataset and the high-resolution imagery used for manual visual interpretation. It can be found that some urban lakes existing in the Sentinel-2 imagery degenerated to bare land. The contrary situation also exists somewhere else.

**Fig. 12:**
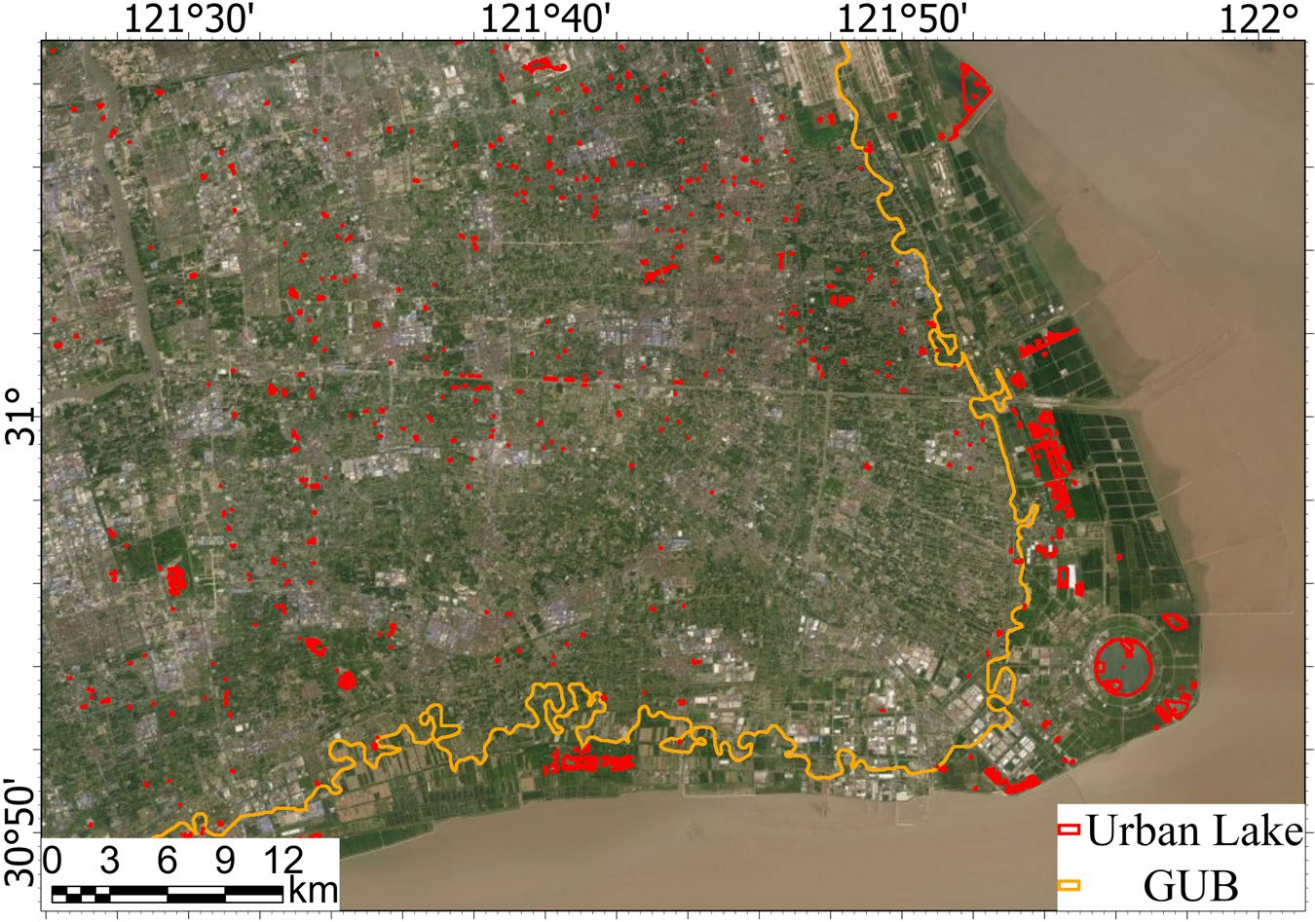
The rapid change of urban boundaries.

Besides the rapid changes of urban lakes, the cities themselves have been expanding dramatically. The adopted GUB reference data were extracted from the images acquired around 2018, which does not represent the extent of urban areas exactly around 2020. Figure 12 illustrates one of the most typical examples of the sprawl of urban boundaries. Though we have manually checked the expansions of urban boundaries of coastal cities, such problems may still exist in inland cities and cause the loss of newly-emerged urban lakes.

**Fig. 13:**
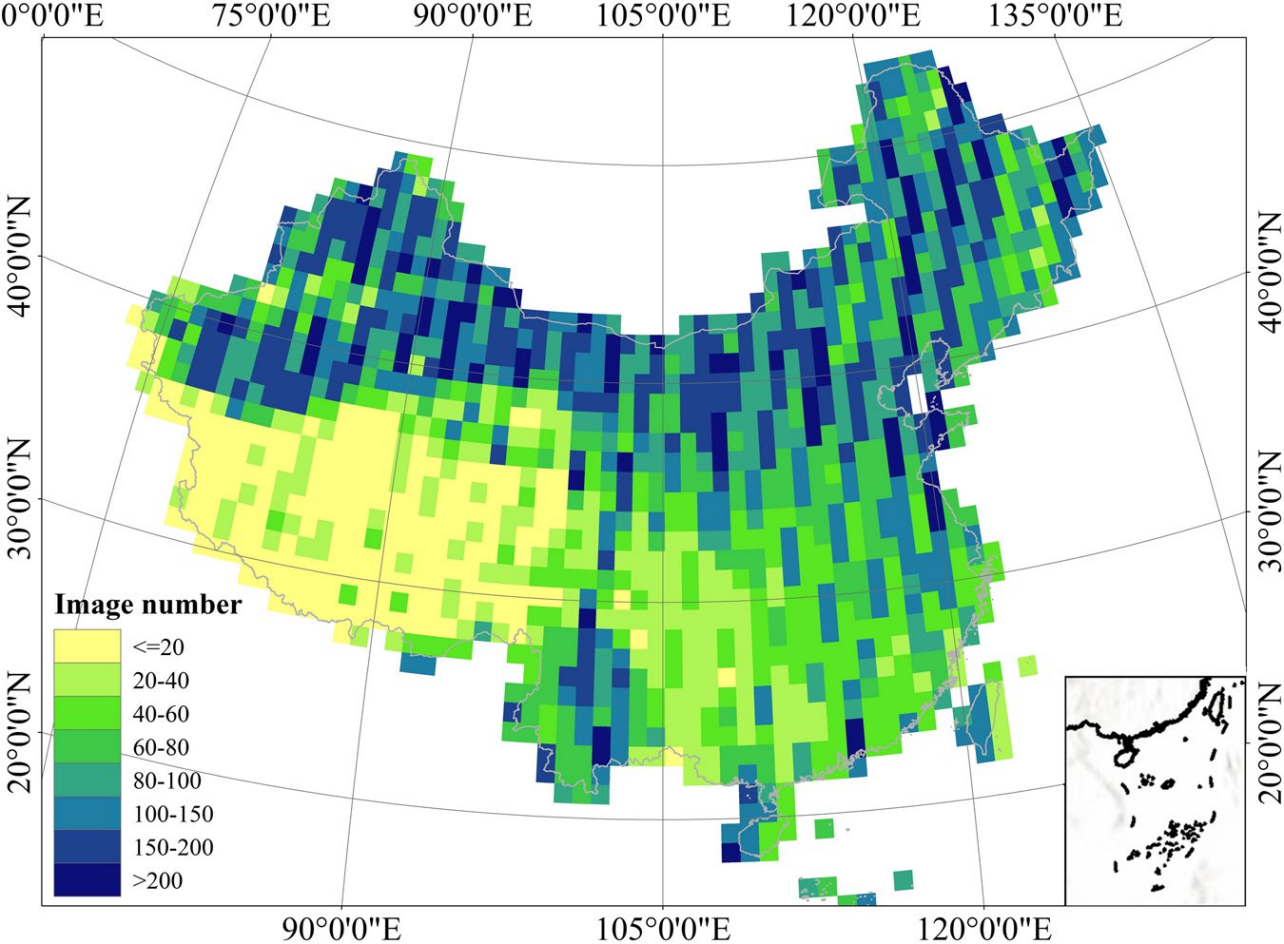
The availability of good quality (cloud cover <20%) images within the selected time windows (2019–2021).

**Fig. 14:**
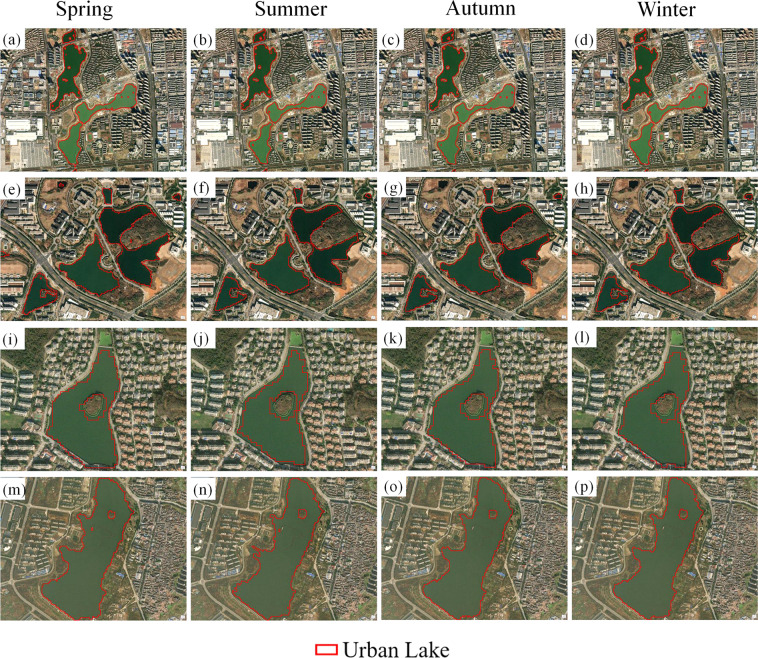
Water inundation areas of urban lake cases in 2020 for different seasons: spring (**a,e,i,m**), summer (**b,f,j,n**), autumn (**c,g,k,o**), and winter (**d,h,l,p**).

High-quality image availability for lake mapping as well as the temporal variations of urban lakes also have inevitable impacts on the mapping results. Figure 13 shows the availabilable count of good-quality (cloud cover <20%) images within the selected time windows (2019–2021). We can see that nearly all of the northern China are covered by more than one hundred of qualified images and most of the eastern and central parts have >50 good images. There are fewer images available in the Qinghai-Tibet region where there are few urban areas in these regions and the climate is dry. Thus, the multi-temporal median composite effectively reduce the influences of cloud cover and shadow. In this study, we aim to produce a static dataset of the circa-2020 urban lakes dataset of China that represent the average status of lake extents. Actually, the inundation areas of urban lakes are rather stable at the intra-annual timescale as urban lake shorelines are usually formed by artificial impervious surfaces and therefore have low seasonal variations (Fig. 14). Furthermore, although the method used in this study to detect urban water bodies is applicable to most cases, the detection of seasonal variation often faces the situation of fewer available images, which make it difficult to guarantee the spectral features of the median imagery to make sure that the proposed fixed thresholds work well.

## Data Availability

The data production algorithms have been introduced in the manuscript, which were conducted in GEE with simple implementation of water index methods. The code which is used to detect urban waterbody in GEE is available with the dataset at the figshare repository (10.6084/m9.figshare.20583558)^70^.
